# Loss of autophagy affects melanoma development in a manner dependent on PTEN status

**DOI:** 10.1038/s41418-021-00746-7

**Published:** 2021-03-04

**Authors:** Mathias T. Rosenfeldt, Jim O’Prey, Colin R. Lindsay, Colin Nixon, Sabine Roth, Owen J. Sansom, Kevin M. Ryan

**Affiliations:** 1 Cancer Research UK Beatson Institute, Glasgow, UK; 2 Comprehensive Cancer Center Mainfranken, Wuerzburg, Germany; 3 Department of Pathology, University of Wuerzburg, Wuerzburg, Germany; 4 Institute of Cancer Sciences, University of Glasgow, Glasgow, UK

**Keywords:** Cancer, Genetics, Cancer models

(Macro)Autophagy is a process that delivers cellular constituents for lysosomal degradation. Autophagy is important in cancer with both pro- and anti-tumourigenic roles being reported [1]. Melanoma is a cancer originating from melanocytes and frequently develops in UV-exposed skin [2]. To examine autophagy’s role in melanoma we utilized a mouse model containing an allele of the *Braf*^*V600E*^ mutation (the signature molecular driver of human melanoma [3]) preceded by a Lox-STOP-Lox cassette [4]. These mice were crossed to animals bearing a floxed allele of *Pten*, which can accelerate the disease, and/or animals carrying floxed alleles of the essential autophagy gene *Atg7* [5, 6]. Recombination of alleles was achieved by topical application of tamoxifen to activate an inducible Cre-recombinase (Cre-ER) under control of the Tyrosinase (*Tyr*) promoter [7]. In *Braf*^*V600E*^ mice wild-type for *Pten* (Tyr-Cre:ER *Pten*^*+/+*^ *Braf*^*V600E/+*^), deletion of *Atg7* (Tyr-Cre:ER *Pten*^*+/+*^ *Braf*^*V600E/+*^ *Atg7*^*−/−*^) significantly accelerated melanoma onset (Fig. 1, Table S1). In contrast, in mice hemizygous for *Pten* (Tyr-Cre:ER *Pten*^*+/−*^ *Braf*^*V600E/+*^) melanoma onset was accelerated, but no difference was observed upon *Atg7* deletion (Fig. 1, Table S2). Immunohistochemistry for ATG7, LC3 and p62/SQSTM1 established the presence/absence of autophagy and positivity for S100 confirmed melanocytic origin of tumour cells (Fig. S1). Our findings show autophagy is dispensable for melanoma growth and might support a barrier function for melanoma development that is compromised in animals hemizygous for *Pten* that eventually lose the remaining wild-type allele during disease progression [5].

**Fig. 1:**
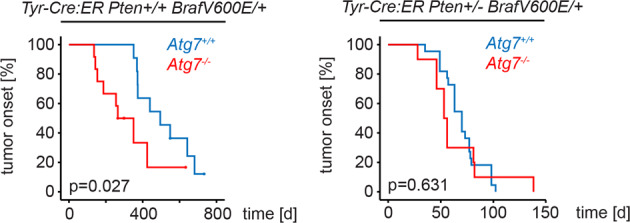
Impact of autophagy deletion on melanoma development. Kaplan–Meyer curves depicting tumour onset of the indicated genotypes. A Log-Rank test was used to compare the tumour onset distribution and *p* < 0.05 was considered statistically significant. Censored animals had to be sacrificed because they reached endpoint criteria or old age without evidence of melanoma.

A potential explanation for our findings might be the connection between autophagy and senescence. Senescence is a terminal cell cycle arrest that serves as a barrier to block malignant progression [8]. Nevi can progress to melanoma if the senescent barrier is breached and downregulation of ATG5 prevents oncogene-induced senescence in primary human melanocytes [9]. As our model develops senescent nevi before invasive melanoma occurs [4], it is conceivable that acceleration of melanoma onset upon *Atg7* deletion is due to a senescence defect. This suggests that *Atg7*-deletion should not have the same impact in the context of *Pten*-deficiency, as PI3K pathway activation via *Pten* deletion is known to abrogate oncogene-induced senescence and contributes to melanoma development [10]. The pro-senescence features of autophagy would then be superfluous and autophagy-deletion would not impact on melanoma onset.

Our results are in contrast to work from Xie and colleagues who found that *Atg7*-deletion prevents tumour formation in the context of *Pten* deletion [11]. A possible explanation for this discrepancy is the different models used. Xie et al. used mice that upon *BrafV600E* activation developed pigmented skin lesions, but failed to progress to invasive melanoma unless combined with loss of PTEN, which would impair senescence from the outset of tumour development [5, 10, 11]. In this case, autophagy loss impairs disease progression by modulating oxidative stress. In contrast, we relied on a different model [4] in which *BRAFV600E* expression alone is sufficient for melanoma development once the senescence barrier is breached at a point during disease progression or, in our case, when combined with loss of an essential autophagy gene. The reason for the different phenotypic effects seen in these two models of melanoma is unknown. It may be that subtle differences in the strategies for gene targeting [12, 13], the use of different Cre lines [7, 14] or differences in the strain background of the animals used in the different studies could all contribute to the differences observed. While this is however speculation, what is clear is that the profound phenotypic differences observed imply significant genetic intricacies that lead to different autophagy dependencies that are affected by *Pten* status in these two mouse models.

It is natural to question how these findings relate to the role of autophagy in human melanoma. Like in many cancers, autophagy is considered tumour suppressive in the transition from benign to malignant disease, but conversely tumour promoting in established melanoma [15, 16]. Our data support a tumour preventive role in the early stages of melanoma development, and as mentioned above, a previous study has shown that ATG5 is down-regulated at this stage of human melanoma resulting in enhanced proliferation and bypass of senescence [9]. Another study also showed that hemizygous loss of *ATG5* occurs during melanoma development [17], but as loss of one allele of *ATG5* would not be predicted to inhibit autophagy, this raises the question whether the driver for this loss is an autophagy-independent effect of ATG5. However, while these caveats are possible, there is clear evidence that accumulation of the autophagic substrate p62/SQSTM1 is pro-tumorigenic [18, 19], suggesting that there is pressure to lose or at least temporarily inactivate autophagy during the progression of this disease.

## Methods

### Animal experiments

Mouse strains were described previously [4–6] and of C57BL/6 background. All experiments were carried out in compliance with UK Home Office regulations (Project license number: P54E3DD25). Melanocyte specific recombination was achieved by topical application of 1 mg/d tamoxifen for five consecutive days on the shaven back to activate an inducible Cre-recombinase under control of the Tyrosinase (Tyr) promotor [7]. Experimental mice were 73 +/− 7d old and of equal gender distribution. Animals were monitored thrice weekly for tumour formation and sacrificed once end point criteria were met (melanoma > = 15 mm, ulceration, significant weight loss, weakness and inactivity). Kaplan–Meier curves represent time to tumour onset. Censored animals had to be sacrificed because they reached endpoint criteria or old age without evidence of melanoma. After euthanasia tissue was excised and fixed in 10 % buffered formalin for 24–48 h at room temperature. Fixed tissue was paraffin embedded and 4 µm sections were prepared for hematoxylin and eosin (HE) staining and immunohistochemistry.

### Immunohistochemistry

The following antibodies were used: ATG7 rabbit monoclonal (Cell Signaling, 8558), S100 rabbit polyclonal (Dako, Z031129), LC3 mouse monoclonal (Nanotools, 5F10), and P62 rabbit polyclonal (Enzo Lifesciences, BML-PW9860). Immunohistochemistry was performed using heat induced epitope retrieval for ATG7 and LC3 with Target Retrieval Solution pH9.0 (Agilent/Dako, K800421-2), for P62 with Target Retrieval Solution pH6.1 (Agilent/Dako, K800521-2) and for S100 with citric acid at pH6.0. ATG7 and LC3 were visualized with SuperVision 2 HRP (mouse/rabbit) (DCS Innovative Diagnostik-Systeme, PD000POL) and p62 and S100 with HiDef Detection™ HRP Polymer System (Cell Marque, 954D-40).

### Statistical analyses

Statistical analyses were carried out using IBM SPSS Statistics version 21 for Windows. A log-rank test was used to determine significance in Kaplan-Meier curves.

## Supplementary information

Supplementary Figure and Table Legends

Supplementary Figure 1

Supplementary Table 1

Supplementary Table 2

## References

[1] Mainz L, Rosenfeldt MT (2018). Autophagy and cancer - insights from mouse models. FEBS J.

[2] Jenkins RW, Fisher DE (2021). Treatment of advanced melanoma in 2020 and beyond. J Invest Dermatol.

[3] Hill VK, Gartner JJ, Samuels Y, Goldstein AM (2013). The genetics of melanoma: recent advances. Annu Rev Genomics Hum Genet.

[4] Dhomen N, Reis-Filho JS, da Rocha Dias S, Hayward R, Savage K, Delmas V (2009). Oncogenic Braf induces melanocyte senescence and melanoma in mice. Cancer Cell.

[5] Dankort D, Curley DP, Cartlidge RA, Nelson B, Karnezis AN, Damsky WE (2009). Braf(V600E) cooperates with Pten loss to induce metastatic melanoma. Nat Genet.

[6] Komatsu M, Waguri S, Ueno T, Iwata J, Murata S, Tanida I (2005). Impairment of starvation-induced and constitutive autophagy in Atg7-deficient mice. J Cell Biol.

[7] Yajima I, Belloir E, Bourgeois Y, Kumasaka M, Delmas V, Larue L (2006). Spatiotemporal gene control by the Cre-ERT2 system in melanocytes. Genesis.

[8] Michaloglou C, Vredeveld LC, Soengas MS, Denoyelle C, Kuilman T, van der Horst CM (2005). BRAFE600-associated senescence-like cell cycle arrest of human naevi. Nature.

[9] Liu H, He Z, von Rutte T, Yousefi S, Hunger RE, Simon HU (2013). Down-regulation of autophagy-related protein 5 (ATG5) contributes to the pathogenesis of early-stage cutaneous melanoma. Sci Transl Med.

[10] Vredeveld LC, Possik PA, Smit MA, Meissl K, Michaloglou C, Horlings HM (2012). Abrogation of BRAFV600E-induced senescence by PI3K pathway activation contributes to melanomagenesis. Genes Dev.

[11] Xie X, Koh JY, Price S, White E, Mehnert JM (2015). Atg7 overcomes senescence and promotes growth of BrafV600E-driven melanoma. Cancer Discov.

[12] Dankort D, Filenova E, Collado M, Serrano M, Jones K, McMahon M (2007). A new mouse model to explore the initiation, progression, and therapy of BRAFV600E-induced lung tumors. Genes Dev.

[13] Mercer K, Giblett S, Green S, Lloyd D, DaRocha Dias S, Plumb M (2005). Expression of endogenous oncogenic V600EB-raf induces proliferation and developmental defects in mice and transformation of primary fibroblasts. Cancer Res.

[14] Bosenberg M, Muthusamy V, Curley DP, Wang Z, Hobbs C, Nelson B (2006). Characterization of melanocyte-specific inducible Cre recombinase transgenic mice. Genesis.

[15] Corazzari M, Fimia GM, Lovat P, Piacentini M (2013). Why is autophagy important for melanoma? Molecular mechanisms and therapeutic implications. Semin Cancer Biol.

[16] Rahmati M, Ebrahim S, Hashemi S, Motamedi M, Moosavi MA (2020). New insights on the role of autophagy in the pathogenesis and treatment of melanoma. Mol Biol Rep..

[17] Garcia-Fernandez M, Karras P, Checinska A, Canon E, Calvo GT, Gomez-Lopez G (2016). Metastatic risk and resistance to BRAF inhibitors in melanoma defined by selective allelic loss of ATG5. Autophagy.

[18] Ellis RA, Horswell S, Ness T, Lumsdon J, Tooze SA, Kirkham N (2014). Prognostic impact of p62 expression in cutaneous malignant melanoma. J Invest Dermatol.

[19] Karras P, Riveiro-Falkenbach E, Canon E, Tejedo C, Calvo TG, Martinez-Herranz R (2019). p62/SQSTM1 fuels melanoma progression by opposing mRNA decay of a selective set of pro-metastatic factors. Cancer Cell.

