# Supplementary material for: Loss of autophagy affects melanoma development in a manner dependent on PTEN status
Source: Cell Death Differ. 2021 Mar 4;28(4):1437–9. doi: 10.1038/s41418-021-00746-7 (PMC8027884; doi:10.1038/s41418-021-00746-7)
Supplement: Supplementary file 1 — Supplementary Figure and Table Legends [file 41418_2021_746_MOESM1_ESM.docx]

**Supplementary Figure and Table Legends**

**Supplementary Figure 1 – Immunohistochemistry establishes the presence/absence of autophagy and melanocytic origin of tumour tissue.** Representative histological stainings as indicated of tumour tissue from the specified mouse cohorts. A punctate LC3 staining pattern and absent / weak p62/SQSTM1 expression confirm the presence of autophagy in ATG7 proficient animals. Strong homogenous cytoplasmic LC3 staining and accumulation of p62/SQSTM1 indicate the absence of autophagy in ATG7 deficient mice. S100 expression establishes melanocytic origin of tumour cells. Scale bars represent 20µm. Boxed inserts are magnified areas of the respective panel.

**Supplementary Table 1 – Cohort composition for the indicated genotypes.** The table indicates male to female ratio and states the number of tumour bearing mice vs in “()” the number of total mice for a given gender. The mean tumour onset [d] +/- SEM of animals is also shown.

**Supplementary Table 2 – Cohort composition for the indicated genotypes.** The table indicates male to female ratio and states the number of tumour bearing mice vs in “()” the number of total mice for a given gender. The mean tumour onset [d] +/- SEM of animals is also shown.
