# Supplementary figures and images for: Loss of autophagy affects melanoma development in a manner dependent on PTEN status
Source: Cell Death Differ. 2021 Mar 4;28(4):1437–9. doi: 10.1038/s41418-021-00746-7 (PMC8027884; doi:10.1038/s41418-021-00746-7)

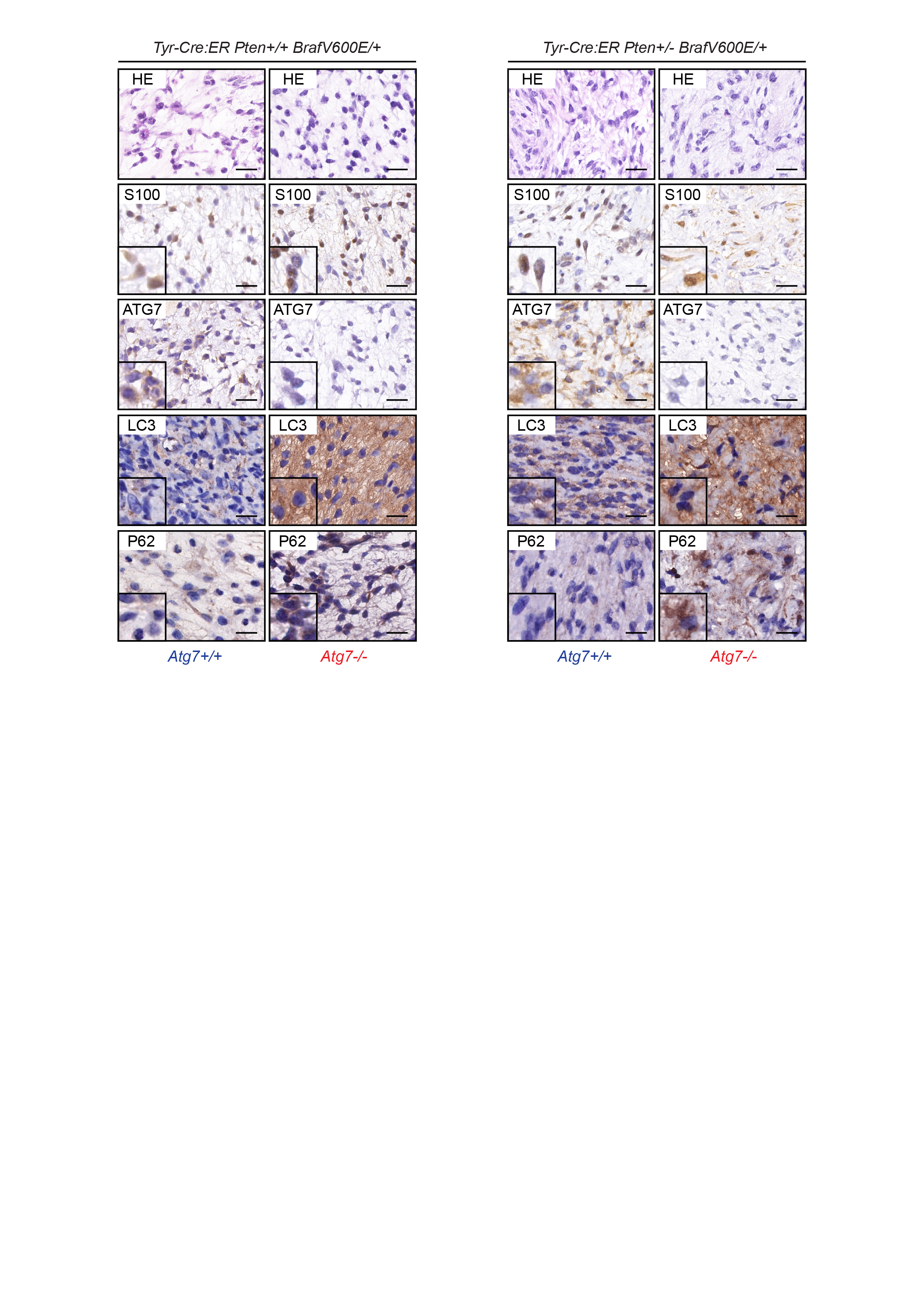

Supplement: Supplementary file 2 — Supplementary Figure 1 [file 41418_2021_746_MOESM2_ESM.png]
