# Supplementary material for: Loss of autophagy affects melanoma development in a manner dependent on PTEN status
Source: Cell Death Differ. 2021 Mar 4;28(4):1437–9. doi: 10.1038/s41418-021-00746-7 (PMC8027884; doi:10.1038/s41418-021-00746-7)
Supplement: Supplementary file 3 — Supplementary Table 1 [file 41418_2021_746_MOESM3_ESM.docx]

*Tyr-Cre:ER Pten+/+ BrafV600E/+*

|  | **male / female**  **events (total)** | **mean onset [d]**  **+/- SEM** |
| --- | --- | --- |
| **Atg7+/+** | 7 (8) / 2 (3) | 517 +/- 43 |
| **Atg7-/-** | 4 (7) / 4 (5) | 330 +/- 54 |
