# Supplementary material for: Loss of autophagy affects melanoma development in a manner dependent on PTEN status
Source: Cell Death Differ. 2021 Mar 4;28(4):1437–9. doi: 10.1038/s41418-021-00746-7 (PMC8027884; doi:10.1038/s41418-021-00746-7)
Supplement: Supplementary file 4 — Supplementary Table 2 [file 41418_2021_746_MOESM4_ESM.docx]

*Tyr-Cre:ER Pten+/- BrafV600E/+*

|  | **male / female**  **events (total)** | **mean onset [d]**  **+/- SEM** |
| --- | --- | --- |
| **Atg7+/+** | 9 (9) / 13 (13) | 70 +/- 4 |
| **Atg7-/-** | 7 (7) / 3 (3) | 64 +/- 10 |
